# The density of electronic gambling machines and area-level socioeconomic status in Finland: a country with a legal monopoly on gambling and a decentralised system of EGMs

**DOI:** 10.1186/s12889-019-7535-1

**Published:** 2019-08-30

**Authors:** Susanna Raisamo, Arho Toikka, Jani Selin, Maria Heiskanen

**Affiliations:** 1 Department of Public Health Solutions, Alcohol, Drugs and Addictions Unit, National Institute for Health and Welfare, P.O. Box 30, FI-00271 Helsinki, Finland; 2 Faculty of Social Science, University of Helsinki, P. O. Box 54, FI-00014 Helsinki, Finland; 3 Gambling Clinic (The Centre of Excellence on Social Welfare in the Helsinki Metropolitan area), Helsinki, Finland

**Keywords:** Electronic gambling machine (EGM), Density, Socioeconomic status (SES), Gambling

## Abstract

**Background:**

Electronic gambling machines (EGMs) are considered a risky form of gambling. Internationally, studies have reported that the density of EGMs tends to be higher in socioeconomically disadvantaged areas than in more advantaged ones. We examined whether this holds true in the Finnish context where a decentralised system of EGMs guarantees wide accessibility to this form of gambling. More precisely, we investigated the association between the density of EGMs and area-level socio-economic status (SES).

**Methods:**

The primary measure was the EGM density, referring to the number of EGMs per 1000 adults. The area-level SES was defined on the basis of the median income of inhabitants, the proportion of unemployment in the area and educational attainment (% of those beyond primary education). Three additional area characteristics were used as control variables in the analyses; the overall population density, economic activity (the number of jobs in the area per employed inhabitant), and the mean age of the inhabitants. Analyses were based on linear regression.

**Results:**

The EGM density was 3.68 per 1000 inhabitants (*SD =* 2.63*).* A lower area-level SES was correlated with a higher EGM density. In further analyses, this effect was mostly explained by the income of the inhabitants. Of the control variables, the population density had no detectable effect on the EGM density while areas with a higher mean age of the inhabitants, as well a higher density of jobs, had more EGMs.

**Conclusions:**

EGMs are unequally located in Finland, with more EGMs located in socio-economically less advantaged areas. The higher machine density in areas of social disadvantage is not in line with the aim of the Finnish gambling policy, which is to prevent and reduce harm caused by gambling. Changes in policy are required, especially with regard to the decisions on the placement of EGMs. This should not be made solely by gaming operators and/or from fiscal perspectives.

## Background

Electronic gambling machines (EGMs) are considered one of the most harmful forms of gambling [1, 2]. This is mainly due to specific features of EGMs such as the rapid speed of play, high payback percentage and near-win events. These features encourage players to gamble continuously and are also shown to be relevant to the process of developing gambling-related problems [2, 3]. Given its harmfulness and in order to reduce gambling-related harm, many jurisdictions internationally have already tightened the regulation of the EGMs or are currently considering greater control of machine gambling [4–6].

### A socio-ecological approach to gambling

To date, much of the gambling research literature available has focused on gamblers’ individual characteristics, problem gambling, its risk factors and correlates. A socioecological approach applied to (EGM) gambling and to gambling-related harm [6–8] emphasises that gambling is not solely an individual behavior; it is a complex interplay between an individual and the broader social, physical and political environment in which they live. Fewer investigations have looked at the contextual and/or environmental determinants of gambling. It has been argued, however, that the more accessible gambling opportunities are in an area or community, the more people will choose to gamble, and this is considered as a concern mainly because increased consumption of gambling has also been linked with increased quantity of gambling-related harm [9–11].

When it comes to EGMs, a review study by Vasiliadis et al. [12] found a relationship between a greater density of EGMs and higher gambling participation rates as well as a higher expenditure on gambling. Evidence further suggest that a higher EGM density is associated with higher rates of problem gambling, and on the other hand, higher rates of help-seeking for problem gambling [13]. The EGM density has also been shown to be highest in socioeconomically disadvantaged areas [6, 14–18]. The concentration of EGMs in socioeconomically disadvantaged areas may lead to harmful patterns of gambling such as risky gambling and thus, increase or instigate gambling harm. The reasons why this might be the case are likely to be multidimensional. It has been presented, for example, that several risk factors of gambling harm are more prevalent in the population living in those areas and that gambling may be used as a coping mechanism to deal with stress and poorer quality of life [6, 19]. The gambling literature has further shown that rates of gambling and problem gambling are higher among individuals with a lower socioeconomic status [20–22].

It is well established that gambling has strong redistributive effects, and state-controlled gambling in particular can be regarded as regressive form of taxation [11, 23, 24]. Gambling is also a very concentrated activity [11, 25, 26]; in Finland half of the revenue comes from 5% of gamblers, about 23% of the gambling revenue comes from problem gamblers, and about 30% of the gambling revenue comes from those in precarious social and financial positions (unemployed, or retired due to age or illness) [27]. Moreover, EGM gambling seems to be especially concentrated; it has been estimated in Australia that problem gamblers contribute about 40% of the total EGM revenue [25]. A recent study on EGM spending in France and Quebec shows that the revenue share of problem gamblers was 41% and 76%, respectively [26]. The high density of EGMs in socioeconomically deprived areas is thus likely to further contribute to the socioeconomic differentiation of the areas and the concentration of gambling.

### The gambling context in Finland

Gambling in Finland is based on a legal monopoly and is operated by a single state-owned company, Veikkaus Oy. Gambling is ubiquitous; 80% of the population aged 15 to 74 years report having gambled during the past year, and the most popular forms of gambling are weekly lotteries, scratch cards and EGMs. Approximately a third of the gambling population gamble on EGMs, and it is estimated that 21% of weekly EGM gamblers are problem gamblers [28]. Finnish clients seeking help for their gambling problems report that EGMs are the most common form of gambling and also the most problematic mode of gambling. Furthermore, customers of the problem gambling clinic in Finland gamble on EGMs predominantly in land-based venues [29]. Land-based EGMs account for approximately a third of the total share of gambling expenditure in Finland, and in 2016, approximately 582 million euros were lost on land-based EGMs [30].

Under the Lotteries Act, the main aim of the Finnish gambling policy is to prevent and reduce the financial, social and health-related harm caused by gambling. Paradoxically, EGMs are visible and easily accessible throughout the country due to a decentralised system. There are approximately 18,500 EGMs in over 6600 venues including ordinary social venues such as grocery stores, kiosks, gas stations, restaurants, bars and cafés. In addition, about 2600 EGMs can be found in dedicated gaming arcades. Decisions related to the placement of EGMs are made solely by the gaming operator and is based on economic interests. Only the maximum numbers of decentralised EGMs and EGMs in arcades are decreed by the Finnish Government.

In this paper, we aim at exploring the relationship between gaming machine density and the area-level socio-economic status (SES), as measured by the unemployment rate, level of education and median income. Given the previous research in the field, we hypothesised that the machine density would be more pronounced in areas characterised by a greater share of the disadvantaged population. However, previous studies have not been conducted in a similar context with a similar gambling policy system to the one which exists here in Finland. As mentioned above, several factors make Finland a very interesting case. There are neither licenses required when EGMs are placed nor any regulations or geographically demands concerning the location of EGMs. The placement of the EGMs is based solely on the economic considerations of the state monopoly company. Finally, because of the decentralised system the accessibility and availability of the EGMs is likely to be on a higher level in Finland than in jurisdictions studied before, where EGMs are located in arcades, casinos, restaurants or clubs [12, 31]. Thus, Finland offers an interesting case example. A theoretical rationale for the current study lies in the socio-ecological approach, and thus, the findings are discussed in the light of this view.

## Methods

The data for the analyses originated from two sources: EGM location data including the number of decentralised EGMs according to the postcode level in Finland were based on the gaming operator’s (Veikkaus) data. The dataset included 18,460 decentralised EGMs. EGMs located in the Helsinki Casino and other dedicated gaming arcades were not taken into account in the analyses since information on these was not available. Measures describing the area-level socio-economic status (SES) were based on data from Statistics Finland. The data from Statistics Finland is based on open data according to postal codes containing information about the areas’ degree of education, age structure of the population, the median income of households, and jobs per 1000 inhabitants. The data used was available at the post code level. Finnish post code areas are internally reasonably homogenous, and have been used as units in measuring socio-economic status of different areas [32]. However, they are obviously defined for a different purpose, and they are not coherent communities and vary in population and size [33]. Post code areas do not reveal micro-level effects, but they should reveal the socio-ecological exposure to EGMs in daily life reasonably well.

### Measures

#### EGM density

The EGM density was defined as the number of EGMs per 1000 adults and was used as a measure of the relative accessibility of EGMs.

#### Area-level socio-economic characteristics

We were interested in how many EGMs are located in areas with different levels of socio-economic status. We measured the area-level SES by the median income of inhabitants, the proportion of unemployment in the area and educational attainment (% of those who have not attained a primary education). Similar indicators describing area-level socioeconomic status are widely used both in health and gambling research (e.g. [16, 17, 34]).

#### Other area characteristics

In addition to the variables mentioned above, three other area characteristics were included in the analyses as control variables. These were the overall population density, economic activity (defined as the number of jobs in the area per employed inhabitants – to differentiate areas where people commute to and from), and the mean age of the inhabitants in the area. Socio-economically different areas obviously differ in other ways as well, and the aim is to include those factors that could theoretically correlate with both the socioeconomic status and the number of EGMs. The concentration of inhabitants and workplaces control for the type of area. For instance, it could be possible that low-status areas are also densely populated, and thus we will get a clearer picture of the effects of the socioeconomic status of the areas by controlling for those issues with regression models. The design of the analysis is such that the areas are reasonably homogenous - except for those variables and control variables included in the models. Especially, controlling for the number of jobs per population helps in distinguishing the areas. The removal of sparsely populated (less than 1000 inhabitants) areas also helps with this goal.

### Data analysis

First, descriptive analyses (means, standard deviations, minimums, and maximums) were calculated for all variables used in the present study (Table 1.). Altogether, 1006 post code areas with a minimum of 1000 inhabitants and at least 1 EGM were chosen for the analyses. The justification for the criteria was that areas with disproportionately high EGM densities were excluded due to low numbers of inhabitants, such as rural areas, shopping centre areas, and areas with public transportation hubs.

**Table 1:** Descriptive statistics

	Variable	Mean	Standard deviation	Minimum	Maximum
1	EGMs per 1000 inhabitants	3.68	2.63	0.16	21.47
2	Median income, 1000€	33.63	8.57	18.41	73.83
3	Unemployed, %	14.55	5.03	2.71	47.56
4	No degree beyond primary, %	26.13	7.03	9.59	45.95
5	Population	4667.44	3653.70	1001	26,245
6	Area, km2	126.25	376.30	0.41	8430.00
7	Population density, 1000s per km2	0.84	1.67	0.0004	21.71
8	Jobs in area/ employed inhabitants * 100	91.65	96.12	8.18	1267.63
9	Mean age	42.65	4.80	26	55

Linear regression analyses were conducted to examine the relationship between the EGM density and the area-level SES indicators. The EGM density was used as a dependent variable, and altogether, five separate models with different sets of explanatory variables were used to elaborate how their effects changed in different models. Models (1), (2), and (3) (Table 2) report the direct, uncontrolled effects of the socio-economic variables, each in turn. Model (4) combines the three socio-economic indicators and Model (5) is the full or final model with all control variables. Significant effects were reported for *p*-values below 0.05 (two-tailed).

**Table 2:** Regression analysis

	*Dependent variable*				
	EGMs per 1000 inhabitants				
	Model (1)	Model (2)	Model (3)	Model (4)	Model (5)
Median income, 1000€	−0.137^***^ (0.009)			−0.125^***^ (0.012)	−0.064^***^ (0.013)
Unemployed, %		0.141^***^ (0.016)		−0.035 (0.020)	0.001 (0.019)
No primary education, %			0.128^***^ (0.011)	0.060^***^ (0.012)	0.035^*^ (0.015)
Population density, 1000s per km2					−0.045 (0.045)
Jobs in area/ employed inhabitants * 100					0.009^***^ (0.001)
Mean age					0.107^***^ (0.021)
Constant	8.268^***^ (0.301)	1.622^***^ (0.245)	0.324 (0.300)	6.797^***^ (0.757)	−0.424 (1.175)

Note: * = p < 0.05, ** = p < 0.01, *** = *p* < 0.001

Data was accessed using the Statistics Finland’s PX-Web service and the statistical analyses were implemented using the R software package [35, 36].

## Results

Table 1 presents descriptive statistics for the variables used in the present study. The mean EGM density rate was 3.68 per 1000 inhabitants (*SD =* 2.63*)*, ranging from 0.16 to 21.47.

In Table 2, Models (1), (2), and (3) show the direct, uncontrolled effects of the socio-economic variables, each in turn. A lower socio-economic status was correlated with a higher EGM density for each of the studied indicators. The results indicated that areas with lower median incomes had more EGMs, as well as areas with more unemployment. Furthermore, areas with a higher proportion of inhabitants with no primary education also had more EGMs. All individual effects were significant at *p* < 0.001.

Model (4) combines the three socio-economic indicators, and as they are correlated with each other, the observed effects change. The results show that the coefficient for income remains largely unchanged, while the education effect is smaller, and the unemployment effect is non-significant. There were more EGMs in socio-economically less advantaged areas, but this effect is mostly explained by income, and to a lesser degree by the educational level. In other words, comparing areas of similar income and education, knowledge of unemployment does not add further insight.

Finally, in Model (5) with all control variables, the effects of the socio-economic variables are smaller, but remain significant for income and education. However, it does not appear to be the case that low-income areas have high concentrations of EGMs just because the areas have a high density of people and jobs, but a lower SES still predicts more EGMs. For the control variables, the population density has no detectable effect, while areas people commute into have a slightly higher number of EGMs, and areas with a higher mean age of inhabitants also have more EGMs.

## Discussion

Consistent with international literature, our findings showed that EGMs are unequally located also in Finland, with more EGMs located in socio-economically less advantaged areas. This pattern was found for each of the studied area-level SES indicators, and was mostly correlated with the income of the inhabitants. When considering the control variables of the study, it was revealed that the population density had no detectable association on the EGM density. This finding is similar to Wardle et al. [17], suggesting that there must be other factors explaining the EGM density than the size of the population. In Australia, for example, the gaming industry has argued that EGMs are located in places where demand is high [37]. Similar claims have also been presented by the gambling industry in Finland. However, as our study shows, it does not appear to be the case that low-income areas have high concentrations of EGMs just because the areas have a high density of people and jobs.

There is a strong socio-economic gradient in gambling and its related harm [20–22]. People living in disadvantaged areas are more likely to be vulnerable to the adverse consequences of gambling than those living in more affluent areas. It is not entirely clear why this is so. From a public health perspective, it is very important to discuss the complex interaction between the game characteristics, the availability and accessibility of gambling machines as well as broader social and economic risk factors [6–8, 37–40]. In the context of EGMs, for example, a recent study by Yücel et al. [38] points to a neuro-socio-environmental model, where an emphasis is put on the interaction of the design features of EGMs with the features of human neurobiology, cognition, and behaviour across the stages of gambling. The authors argue that EGMs can provide relief from stress and other consequences of disadvantage.

From the gambling policy perspective, a broad range of strategies can be adopted to reduce gambling harm in the population. Policy approaches that may be expected to produce preventive effects to the problem of harms arising especially from the EGMs could involve both regulating structural harmful characteristics of EGMs and reducing the accessibility and density of EGMs [39, 40].

This study has limitations that are worth some further consideration. Previous research has established that gambling expenditure is higher in disadvantaged areas compared to advantage ones [15]. Certainly, linking administrative EGM expenditure data to EGM density data would have given us a clearer picture of utilization of EGMs [37]. Unfortunately, EGM expenditure data were not available for the use of research. Another consideration relates to the participation in online EGMs. EGMs can be legally gambled online in Finland and Finnish gamblers seeking help increasingly report having gambled EGMs online, too [41]. The relationship of socioeconomic status and online EGMs use warrant further investigation. What should also be looked into further is the overall availability of gambling products within areas as well as to what extent people living in certain postal code areas gamble outside their own neighbourhoods. The public health implications of the present study could include, for example, the development of risk mapping tools. In subsequent investigations, using Geographical Information System (GIS) techniques instead of postal code information only could provide a more in-depth understanding of the findings [17, 40], since it is possible that population moves between postcodes or machines are located on the border of postcodes and used by people in adjacent areas. This is difficult to account for unless using GIS. Moreover, because EGMs placed in arcades were not included here, it is possible that the association between EGM density and area-level SES is in fact even stronger than estimated here.

## Conclusions

Our study confirms that EGMs are unequally located in Finland, with more EGMs found in socio-economically less advantaged areas. This finding replicates similar findings found previously, for example, in Australia and the UK. Besides providing evidence from a new jurisdiction, our work has relevance to current gambling policy in Finland. Insofar as the prevention of gambling-related problems is considered an important policy objective, the high machine density in areas of social and economic disadvantage is not in line with this objective. As the results of the present study here indicate, leaving too much discretion to the operators is likely to lead to a situation where economic interests prevail. Decisions on the placement of EGMs should not be based on economic interests alone. It is possible that the high EGM density, high accessibility and high availability made possible by the Finnish decentralised system exacerbate the risk of adverse consequences, and due to the redistributive effects of EGM gambling, it is possible that the high EGM density contributes to the further socio-economic differentiation of the areas.

## Data Availability

Data on area-level socioeconomic status of the population is publicly available from Statistics Finland as an open data service per postal code area. Available at: https://www.stat.fi/tup/paavo/index_en.html. The EGM location data analysed during the current study is available on reasonable request from the corresponding author.

## Abbreviations

EGM: Electronic gaming machine
SD: Standard deviation
SES: Socio-economic status
